# The Use of Hemodialysis in Refractory Hypercalcemia Secondary to Parathyroid Carcinoma

**DOI:** 10.1155/2014/140906

**Published:** 2014-01-21

**Authors:** Huai Heng Loh, Nurain Mohd Noor

**Affiliations:** ^1^Faculty of Medicine and Health Sciences, Universiti Malaysia Sarawak, Lot 77, Seksyen 22, KTLD Jalan Tun Ahmad Zaidi Adruce, 93150 Kuching, Sarawak, Malaysia; ^2^Endocrine Unit, Putrajaya Hospital, Jalan 9, Presint 7, 62250 Putrajaya, Malaysia

## Abstract

Parathyroid carcinoma is a rare cause of hypercalcemia. Hypercalcemic crisis is a medical emergency. Hemodialysis is rarely needed to treat hypercalcaemia. We report a case of refractory hypercalcaemia secondary to parathyroid carcinoma requiring haemodialysis.

## 1. Introduction

Parathyroid carcinoma is a rare cause of hypercalcaemia. Hypercalcaemic crisis is a medical emergency, which can lead to severe consequences involving neurological, cardiovascular, and renal systems [1]. Standard treatment for hypercalcaemia includes saline hydration, bisphosphonates, steroids, calcitonin [2], and the now commonly reported cinacalcet [3]. The use of haemodialysis to effectively lower serum calcium level in the emergency setting is documented as one of the modalities; however, its use is not commonly reported. We hereby report a patient with recurrent parathyroid carcinoma who had to undergo haemodialysis to effectively lower the calcium level before undergoing a repeat operation.

## 2. Case Report

A 27-year-old lady, with recurrent parathyroid carcinoma, presented to a private hospital with generalized body ache. Her calcium level was 5.3 mmol/L. She was adequately resuscitated with saline hydration and intravenous. Zoledronic acid 4 mg was given. Upon transfer to our center two days later, her calcium level had fallen to 3.02 mmol/L. Despite five liters of hydration daily, three days of subcutaneous calcitonin 200 IU b.d. (total daily dose of 8 u/kg), and intravenous Pamidronate 90 mg, her calcium continued to rise to 4.0 mmol/L. Her intact parathyroid hormone (iPTH) level was 156 pmol/L. She subsequently developed supraventricular tachycardia, which did not respond to standard medical therapy. The arrhythmia was believed to be related to her hypercalcaemia as echocardiography was normal, potassium was 3.8 mmol/L and phosphate level was 0.9 mmol/L. As cinacalcet was not readily available, a decision was made for her to undergo urgent dialysis. Normal-heparin haemodialysis was initiated via a femoral catheter using low-calcium dialysate of 1 mmol/L, with blood flow rate (Qb) 200 mL/min and dialysate flow rate (Qd) 500 mL/min. Her potassium level reduced to 2.6 mmol/L and phosphate to 0.22 mmol/L after dialysis. Hypophosphatemia was not corrected; however it increased to 0.55 mmol/L the next day. Hypokalemia was aggressively corrected via intravenous route throughout and after her four-hour haemodialysis, which was otherwise uneventful. Post dialysis, her corrected calcium was lowered to 2.6 mmol/L. She required another session of haemodialysis five days later with concurrent use of subcutaneous calcitonin, saline hydration, and forced diuresis, when her calcium level increased to 3.9 mmol/L. She finally underwent a third parathyroidectomy with modified radical neck dissection immediately after the second session of haemodialysis with calcium level of 2.36 mmol/L.

## 3. Discussion

Parathyroid carcinoma is a rare cause of PTH-related hypercalcemia [4]. Patients with this disorder can present with hypercalcaemic crisis, similar to presentation of primary hyperparathyroidism, although markedly elevated calcium typically above 3.5 mmol/L points towards malignancy [5]. Parathyroid carcinoma tends to be recurrent; therefore, these patients usually have repeated admissions for hypercalcaemia. The treatment of choice for parathyroid carcinoma is surgical removal; however, perioperative management of hypercalcaemia is important to reduce the complications and to optimize the patient for operation.

Calcium level above 3.5 mmol/L is considered hypercalcaemic crisis and needs urgent treatment [2]. Standard medical therapy includes intravenous hydration with isotonic saline, intravenous bisphosphonates, and subcutaneous calcitonin. However, these modalities have their own shortcomings. Hydration alone can only lower calcium by 0.4 mmol/L to 0.6 mmol/L and is usually inadequate as a sole therapy in cases of severe hypercalcaemia [6]. However, it is still the first line therapy to be used, as it is important to restore hydration in patients with hypercalcaemia as they are usually dehydrated [6]. Bisphosphonates take its maximal effect only after 48 to 72 hours of administration with its effect lasting for a few weeks [7]. Therefore, it is more suitable as a maintenance therapy and not the treatment of choice if urgent reduction of calcium is needed. Calcitonin may cause rebound hypercalcaemia after 24 hours and is associated with tachyphylaxis with prolonged usage of 48 to 72 hours [7]. Recently, cinacalcet, a calcimimetic agent, has also been found effective in hypercalcaemia secondary to parathyroid carcinoma [8]. However, cinacalcet is not readily available in our region due to its cost, hence leading to its limited use. Occasionally when calcium is refractory to these standard treatments, or when urgent reduction of calcium is needed, especially when there is neurological or cardiac involvement, haemodialysis is effective in reducing calcium level over a short period of time while awaiting more definitive therapy [2].

So far, there is no consensus on the guideline of haemodialysis in hypercalcaemia. The report of this modality in the literature review is surprisingly scarce. Different institutions have used different duration of haemodialysis, ranging from one to five hours. Camus et al. suggested a three-to-four-hour haemodialysis session with calcium-free dialysate to effectively reduce calcium level in an emergency setting [1].

Haemodialysis should be started after adequate hydration, as these patients tend to be hypovolemic due to hypercalcaemia. Starting haemodialysis without adequate restoration of volume status can lead to haemodynamic instability with hypotensive episodes.

The diffusion of calcium during haemodialysis depends on the calcium gradient between the serum concentration and the dialysate concentration [9]. The use of calcium dialysate less than 1.25 mmol/L has been found to have a potential of negative calcium balance [9], hence is likely to be more effective in patients with hypercalcaemic crisis. Many institutions chose calcium-free dialysate during haemodialysis of patients with hypercalcaemic crisis [1, 10–12]. However, the use of low-calcium dialysate <1 mmol/L, and even normal-calcium dialysate of 1.5 mmol/L seem to be equally effective [13–15]. Moreover, as calcium ions play a major role in cardiac contractility, excessive shift in calcium gradient may lead to haemodynamic instability. A larger decline in blood pressure may be seen with lower calcium-containing dialysate although it could be contributed by underestimation of dehydration or inadequate fluid administration prior to dialysis [1, 16].

Due to phosphaturic effect of hyperparathyroidism, these patients are usually hypophosphatemic at presentation. This can be worsened with dialysis, as seen in our patient, as commercially available dialysate for patients on long-term renal replacement therapy is commonly phosphorus-free. Hypokalemia is also common after dialysis as patients with hypercalcaemia secondary to hyperparathyroidism usually have normal or only slight renal impairment. Hence, the correction of these electrolyte imbalances should be looked into during and after dialysis as severe hypokalemia and hypophosphatemia can lead to detrimental cardiac events. Some suggest the use of phosphorus-enriched dialysate [13, 17], while others have used intravenous phosphorus for correction of hypophosphatemia [18].

## 4. Conclusion

Hypercalcaemia is a life-threatening condition and should be managed immediately. Haemodialysis is not the first line treatment for hypercalcaemia; however, it offers an extra option when patients are refractory to standard therapies or if urgent reduction of calcium is needed. Both low-calcium dialysate and calcium-free dialysate are effective but the latter might be associated with hypotensive episodes. Potassium and phosphate correction should be looked into to prevent further cardiac complications.
